# A46 PER-ORAL ENDOSCOPIC MYOTOMY ALLEVIATES HELICAL STENOSIS OF A GASTRIC CONDUIT POST-ESOPHAGECTOMY: A CASE REPORT

**DOI:** 10.1093/jcag/gwae059.046

**Published:** 2025-02-10

**Authors:** S X Jiang, A Dashti, C Thilakanathan, R Trasolini

**Affiliations:** The University of British Columbia Faculty of Medicine, Vancouver, BC, Canada; The University of British Columbia Faculty of Medicine, Vancouver, BC, Canada; The University of British Columbia Faculty of Medicine, Vancouver, BC, Canada; Vancouver General Hospital, Division of Gastroenterology, University of British Columbia, Vancouver, BC, Canada

## Abstract

**Background:**

Gastric conduit dysfunction is a common post-operative complication of esophagectomy. Established treatments include pyloric botox, dilatation, pyloromyotomy, and most definitively, surgical revision. We present a case of gastric conduit dysfunction that was refractory to surgical management.

**Aims:**

To present a case of gastric conduit dysfunction due to helical stenosis; to bring awareness to this rare condition and novel application of endoscopic myotomy.

**Methods:**

Case report and literature review.

**Results: Case Report:**

A 69-year-old male underwent Ivor Lewis esophagectomy for esophageal adenocarcinoma, complicated by significant post-operative dysphagia and regurgitation, requiring complete jejunostomy feeding. Barium swallow showed obstruction at the hiatus and torsion of the gastric conduit proximal to the pylorus. Multiple gastroscopies similarly noted torsional stenosis in the gastric conduit and excluded a gastroesophageal anastomotic stricture. Over several months, he underwent progressive interventions with minimal symptomatic improvement: botox injection to the pylorus, endoscopic balloon dilatation to 20mm, laparoscopic crural revision, and laparotomy with enlargement of the diaphragmatic hiatus and surgical pyloromyotomy. Finally, at 8 months post-esophagectomy, the patient underwent tunneled full-thickness endoscopic myotomy to 3cm of helical stenosis in the gastric conduit with noted release and no complications. Following this, the patient has consumed solid meals without issue.

**Literature Review:**

Gastric conduit dysfunction, encompassing delayed emptying, occurs in 15- 30% of patients post-esophagectomy and can lead to debilitating dysphagia, reflux, malnutrition, and aspiration. Most patients respond to pyloric interventions such as botox, balloon dilatation and pyloromyotomy. Limited case series have described 2-5% of patients with refractory symptoms; one proposed cause was twisting of the gastric conduit such that outflow is obstructed. This scenario is anatomically akin to helical stenosis following laparoscopic sleeve gastrectomy, for which gastric per-oral endoscopic myotomy (POEM) has shown early success. Given exhaustion of surgical management, a similar approach was undertaken in the above patient; we report the first case of a twisted gastric conduit treated with POEM.

**Conclusions:**

With advances in third space endoscopy, surgery is no longer the only option for refractory stenosis in carefully selected patients. In the rare case of severe gastric conduit dysfunction due to helical stenosis, endoscopic myotomy may be a safe and effective treatment.

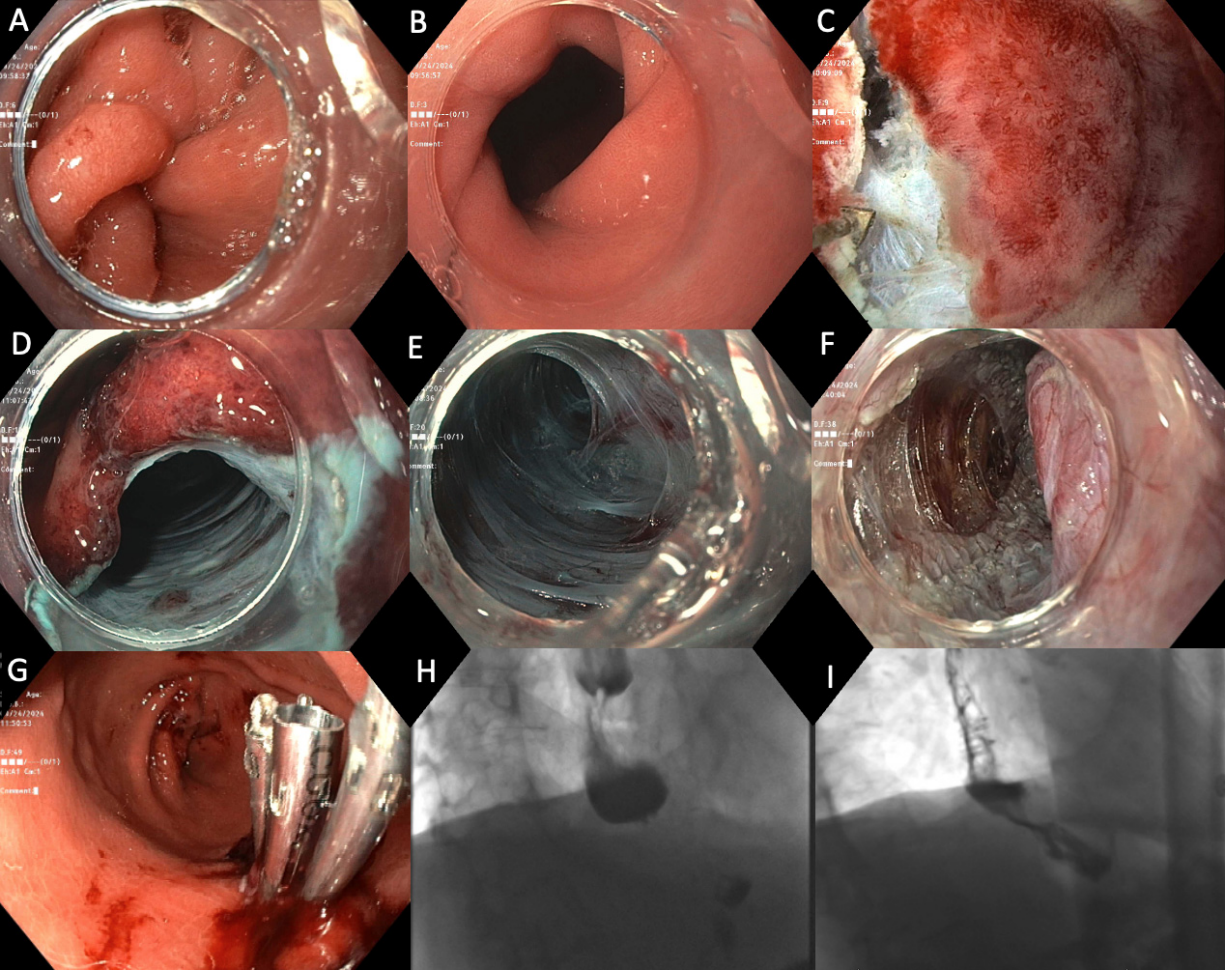

A. Helical stenosis of the gastric conduit proximal to the pylorus. B. Lumen of the helical stenosis. C. Creation of mucosotomy. D. Submucosal tunnel created. E. Tortuosity within tunnel. F. Full thickness myotomy with exposure of the serosal layer in area of stenosis. G. Mucosotomy closed with through-the-scope clips. H. Pre-procedure barium swallow with hold up of contrast at the helical stenosis. I. Post-procedure barium swallow with passage of contrast.

**Funding Agencies:**

None

